# Evaluation of Targeted Agents for Advanced and Unresectable Hepatocellular Carcinoma: A Network Meta-Analysis

**DOI:** 10.7150/jca.32828

**Published:** 2019-08-19

**Authors:** Tao Guo, Pengpeng Liu, Jian Yang, Ping Wu, Baiyang Chen, Zhisu Liu, Zhen Li

**Affiliations:** 1 Department of Hepatobiliary and Pancreatic Surgery, Department of General Surgery, Zhongnan Hospital of Wuhan University, Wuhan 430071, P.R. China.; 2 School of Nursing, Huanggang Polytechnic College, Huanggang, 438002, P.R. China.

**Keywords:** targeted agent, hepatocellular carcinoma, network meta-analysis

## Abstract

**Objective:** To evaluate different targeted anticancer agents for patients with advanced or unresectable hepatocellular carcinoma (HCC) based on network meta-analysis.

**Methods:** Literature retrieval was conducted in globally recognized databases, namely, MEDLINE, PMC, EMBASE and Cochrane Central to find relevant randomized controlled trials (RCTs). Relative parametric data, including overall survival (OS), progression-free survival (PFS) and adverse event (AE), were quantitatively pooled and estimated based on the Bayesian theorem. The values of the surface under the cumulative ranking (SUCRA) probabilities regarding each parameter were calculated and ranked. Node-splitting analysis was performed to test the inconsistency of the main results, and publication bias was assessed by examining funnel-plot symmetry.

**Results:** After a detailed review, 31 RCTs containing 20 different agents or combinations were finally included for network meta-analysis. For patients without previously systematic treatments, lenvatinib had the best clinical effects on OS (SUCRA, 0.22), and apatinib was superior regarding PFS (SUCRA, 0.41) and AE (SUCRA, 0.15). For patients who received previously targeted agents therapies, regorafenib exhibited the superior clinical effects on OS (SUCRA, 0.42) and PFS (SUCRA, 0.30), while codrituzumab showed the greatest safety benefit on AE (SUCRA, 0.75). Moreover, node-splitting analysis and funnel-plot symmetries illustrated no inconsistency or obvious publication bias in the current study.

**Conclusions:** According to current evidence, lenvatinib and apatinib had superior clinical effects for patients without previously systematic treatments, and regorafenib seemed to be more suitable for patients with previously targeted agent therapies. However, our conclusions still need more statistical validations, and more high-quality trials are expected.

## Introduction

Hepatocellular carcinoma (HCC) is still one of the most common malignancies worldwide and the third leading cause of cancer-related death globally, although its management continues to develop 1- 4. HCC usually occurs in the setting of liver cirrhosis due to chronic hepatitis B virus (HBV) or hepatitis C virus (HCV) infections, alcohol consumption, non-alcoholic steatohepatitis, or diabetes, and early intervention is difficult due to unobvious clinical symptoms 5- 7. Therefore, many patients are diagnosed with advanced or unresectable HCC at the first detection with limited therapeutic options and poor prognosis 8. Systematic therapy seemed to be effective, but non-target effects may cause severe autologous injury 9. Thus, novel target anticancer agents for advanced HCC are urgently needed.

In the last 2 decades, increasingly more molecular mechanisms and various signalling pathways in HCC have been discovered, and relative targeted agents were developed to provide a new therapeutic method for advanced HCC 10. Sorafenib is the first targeted agent approved as first-line therapy for patients with advanced hepatocellular carcinoma in the last decade 11. It is a multikinase inhibitor of vascular endothelial growth factor (VEGF) receptor and platelet-derived growth factor (PDGF) receptor and can translate into clinical benefits 12- 13. Subsequently, other targeted agents based on other target molecules or highly selective agents were also applied for advanced HCC in clinic 14- 15. In addition, even multiple second-line targeted agents were reported for patients with previous poor systematic treatment or with sorafenib-resistance or -intolerance 16- 17. Thus far, current therapeutic options of targeted agents for advanced or unresectable HCC lack uniform standards. Moreover, the clinical choices for advanced HCC patients may also need to consider relevant previous conditions. Based on these facts, we attempted to conduct a comprehensive quantitative comparison to evaluate different targeted agents for advanced or unresectable HCC based on a network meta-analysis. The current study also undertook the burden to provide relative clinical evidence for decision-making.

## Methods

### Literature Search and Retrieval

Our review was initialized according to previously established Preferred Reporting Items for Systematic Reviews and Meta-Analyses (PRISMA) guidelines 18 and it was pre-registered with PROSPERO with ID CRD42019121081. Literature retrieval was conducted in global recognized electronic databases (namely, MEDLINE, PMC, EMBASE and Cochrane Central) to achieve the authority of raw data. MeSH terms individually or in combination were used to address relative trials (example search strategy in MEDLINE is presented in Supplementary Table S1). Relevant trails were searched from inception to March 2019 without publication status restriction although full English texts need to be detailed reviewed if raw data was needed.

### Inclusion and Exclusion Criteria

Two researchers independently reviewed the title and the abstract of each assay to select those for further screening that met following items: (1) randomized controlled trials (RCTs); (2) trials focused on targeted agents for advanced or unresectable HCC; (3) application of targeted agent was the only intervention; and (4) studies providing at least one available parameter of interests. Meanwhile, the following items were defined as exclusion criteria: (1) observational studies; (2) no available parametric data reported; (3) reviews, comments, case reports or study protocols; (4) studies focusing on basic science; (5) trials without sufficient follow-up; and (6) mixed HCC stages or repeated reports.

### Data Extraction and Quality Assessment

A pre-designed form was used to record general information and intervention-related characteristics. In the current study, we aimed to evaluate different targeted agents for advanced or unresectable HCC. Thus relevant parametric data of efficacy and safety were selected for pooled estimation. Due to the short survival period of advanced HCC, 1-year overall survival (OS) and progression-free survival (PFS) were estimated as clinical efficacy, and adverse event (AE) was assessed as safety. Meanwhile, samples with or without previously systematic therapy were separately compared. Moreover, for those papers presenting only survival curves, Engauge Digitizer (version 4.1) was used to extract raw data of abstinence proportions 19- 20.

On the other hand, since we only included RCTs for the current study, Cochrane Risk of Bias assessment tool was used to assess the bias risk of individual studies in 6 different aspects 21. Additionally, graphic summaries of bias was plotted by using Review Manager Software (version 5.3). The raw data extraction and bias risk assessment were independently conducted by two investigators. Any disagreements were resolved by a group discussion with all team members.

### Statistical Analysis

In the current study, we focused on the clinical values of different targeted agents for HCC. Thus, indirect pooled estimation of included agents was conducted to make comprehensive network comparesons based on the Bayesian theorem. It combines direct and indirect information to quantitatively estimate multiple interventions to comprehensively address the superior rank 22- 23. The values of the surface under the cumulative ranking (SUCRA) probabilities are presented to clarify the pros and cons of different agents. Based on the SUCRA values, different ranks of included agents are presented, and the superior agent would be determined with the highest SUCRA probabilities in the best rank regarding each parameter. In addition, publication bias was assessed by funnel-plot symmetry. Meanwhile, according to the closed loops in network connections, node-splitting analysis was also performed and showed no statistical inconsistency at *P* > 0.05 24. Convergence was assessed to calculate the Potential Scale Reduction Factor (PSRF), and values were limited to 1 to complete the calculation. The automated software Aggregate Data Drug Information System (ADDIS, version 1.16) and Stata software package (version 12.0) were used for the network-pooled estimation.

## Results

### Study Selection and Characteristics

**Figure 1:**
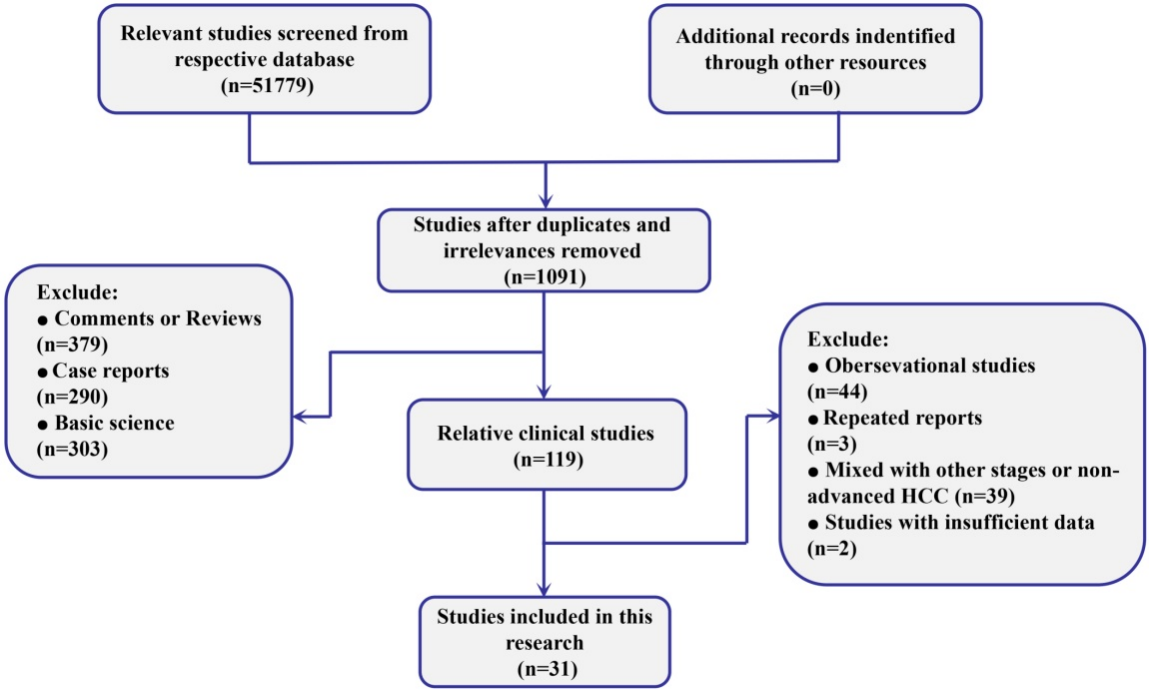
Flow diagram of the process of selecting studies for current network meta-analysis.

The searches identified 51779 records, of which 1091 were considered relevant clinical studies after titles and abstracts were reviewed. Eventually, based on the review of full texts, 31 trials containing 13023 patients were included for quantitative pooled estimation (Figure 1). All of them were 2-arm trials reported from 10 different regions. Among them, 22 of 31 trials were performed based on the samples without any previous systematic treatment, and the other 9 were based on patients treated with previously targeted agents. Twenty different targeted agents or combinations were included for analysis and both placebo and no treatment were regarded as negative control (NC) (Supplementary Table S2). On the other hand, for quality assessment, more than half of included trials were conducted with randomized allocation with concealment. Moreover, most of the trials were based on double-blind processes. In general, the overall quality was well presented (Supplementary Figure S1).

### Main Results of Network Meta-analysis

**Figure 2:**
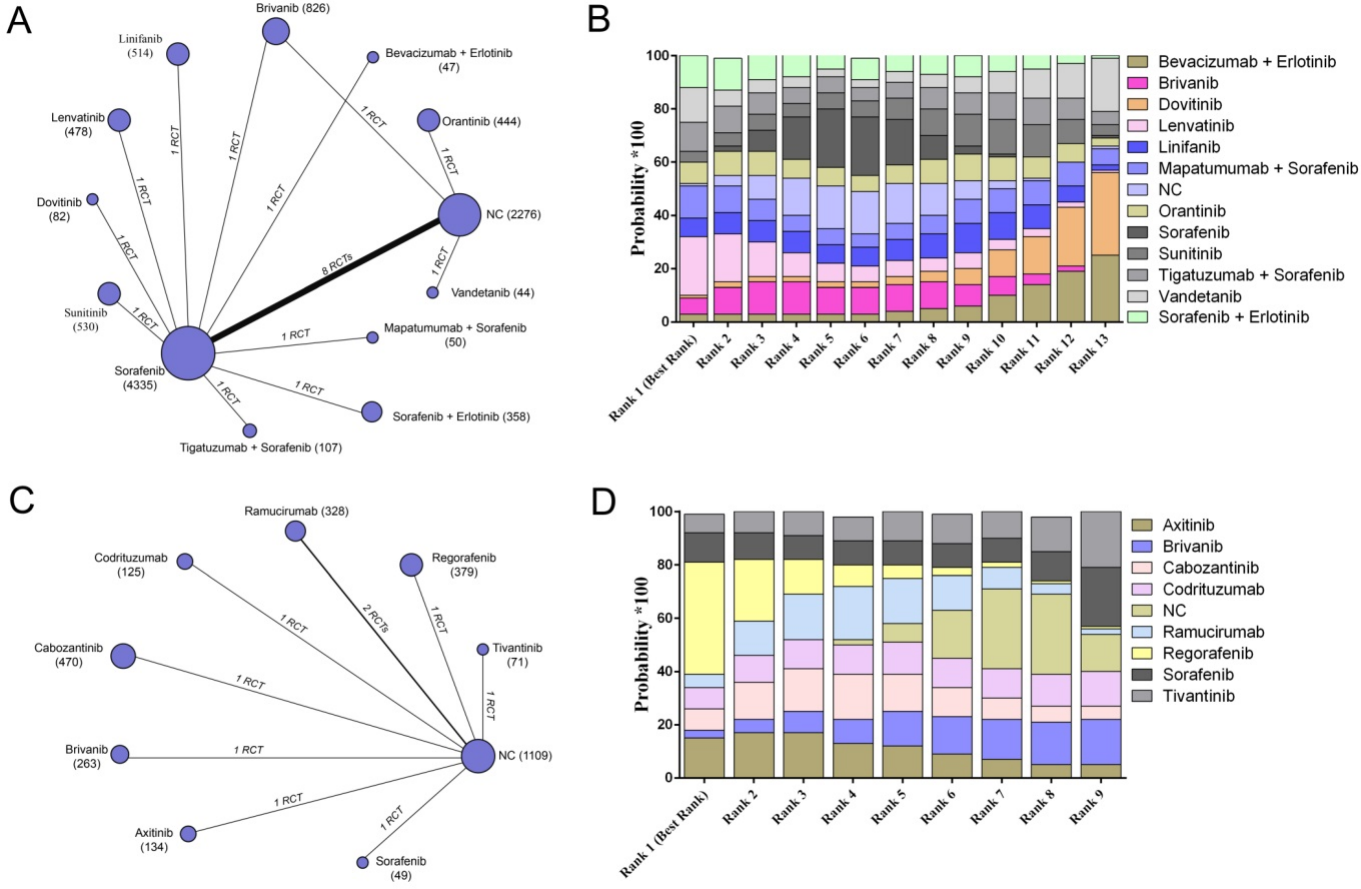
The network evaluation of included agents regarding overall survival. (A) Network connections of all of the included trials for patients without previous systematic treatments; (B) SUCRA values and ranks of included agents for patients without previous systematic treatments; (C) Network connections of all of the included trials for patients with previous systematic treatments; (D) SUCRA values and ranks of included agents for patients with previous systematic treatments. The numbers on the line indicate the quality of studies compared with every pair of strategies, which are also represented by the width of the lines. Additionally, the sizes of the areas of the circles indicate the respective sample sizes. NC, negative control.

We conducted network meta-analysis to evaluate the efficacy and safety of all included targeted agents regarding overall survival, progression-free survival, and adverse events. For overall survival, 20 trials containing 10091 patients reported relative parametric data (Figure 2A). After pooled estimation, we discovered that lenvatinib had the highest probability of achieving the best 1-year overall survival for patients without previous systematic treatment (SUCRA, 0.22) (Figure 2B). Nine trials reported 8 different agents or combinations and evaluated patients with previous targeted drugs therapy (Figure 2C). The results indicated that regorafenib possessed the highest probability of achieving the best 1-year overall survival (SUCRA, 0.42) (Figure 2D) (Supplementary Table S3).

**Figure 3:**
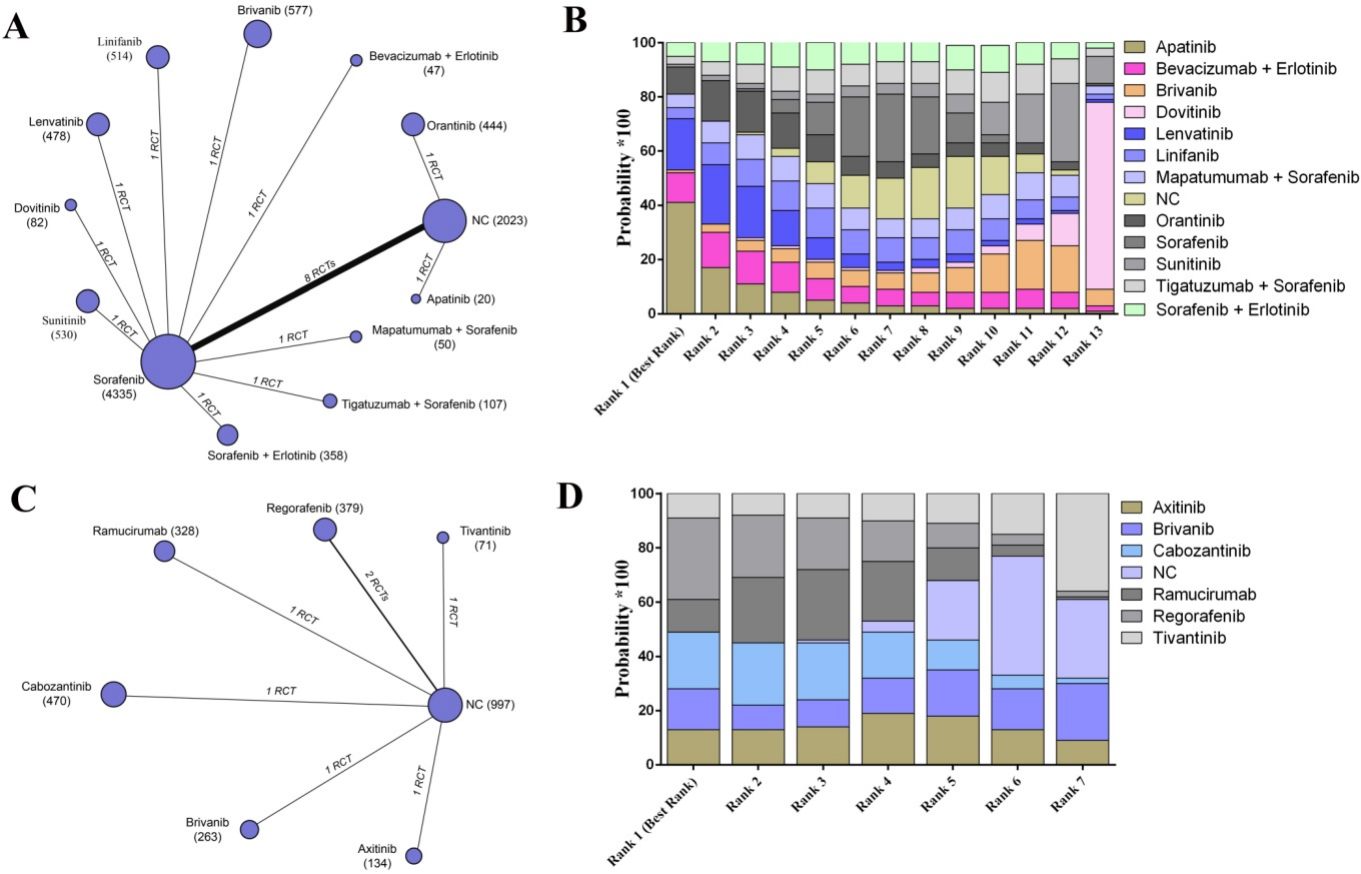
The network evaluation of included agents regarding progression-free survival. (A) Network connections of all of the included trials for patients without previous systematic treatments; (B) SUCRA values and ranks of included agents for patients without previous systematic treatments; (C) Network connections of all of the included trials for patients with previous systematic treatments; (D) SUCRA values and ranks of included agents for patients with previous systematic treatments. The numbers on the line indicate the quality of studies compared with every pair of strategies, which are also represented by the width of the lines. Additionally, the sizes of the areas of the circles indicate the respective sample sizes. NC, negative control.

For the evaluation of progression-free survival, 19 trials including 13 agents or combinations and reported the relative parametric data based on the samples without previous targeted agent treatment (Figure 3A). According to the results, it was detected that apatinib may have the best clinical efficacy on enhancing 1-year progression-free survival in the best rank for patients without previously systematic treatments (SUCRA, 0.41) (Figure 3B). Moreover, regorafenib possessed the highest probability of revealing the best progression-free survival among the patients who received previous systematic therapies based on an established network from 7 trials (SUCRA, 0.30) (Figure 3C- D) (Supplementary Table S4).

**Figure 4:**
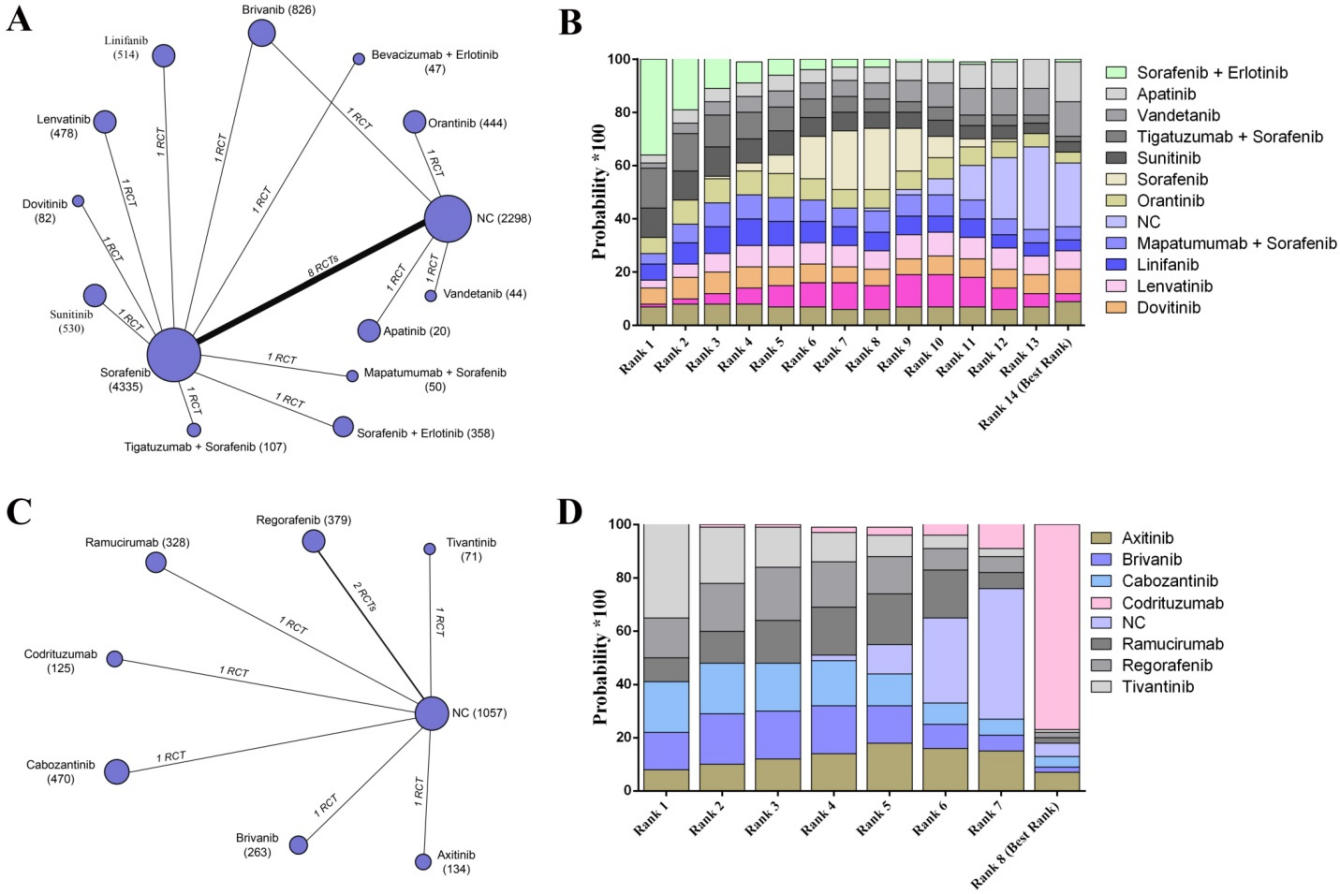
The network evaluation of included agents regarding adverse event. (A) Network connections of all of the included trials for patients without previous systematic treatments; (B) SUCRA values and ranks of included agents for patients without previous systematic treatments; (C) Network connections of all of the included trials for patients with previous systematic treatments; (D) SUCRA values and ranks of included agents for patients with previous systematic treatments. The numbers on the line indicate the quality of studies compared with every pair of strategies, which are also represented by the width of the lines. Additionally, the sizes of the areas of the circles indicate the respective sample sizes. NC, negative control.

To assess the safety of different targeted agents, the data of adverse event were pooled estimates. For the participants without previous targeted agent therapy, 20 trials reported relative raw data, and the results indicated that patients without any agents had the lowest adverse rate (SUCRA, 0.22) followed by apatinib (SUCRA, 0.15) (Figure 4A- B). On the other hand, codrituzumab had the greatest safety effects on reducing adverse events for patients receiving previous targeted agents therapies based on the established network from 8 trials (SUCRA, 0.75) (Figure 4C- D) (Supplementary Table S5).

### Publication Bias and Data Consistency

**Figure 5:**
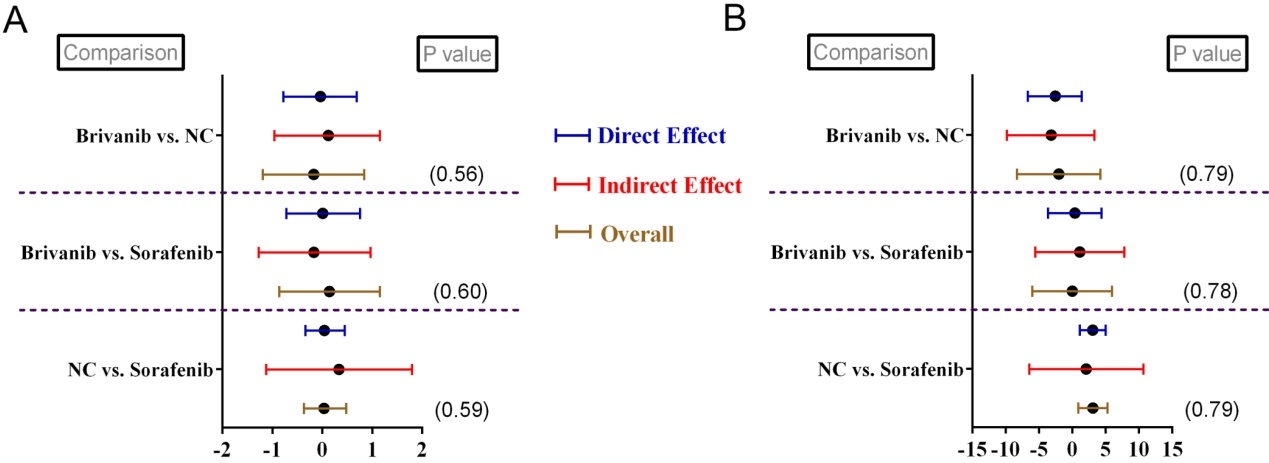
Node-splitting analysis for patients without previously systematic treatment regarding (A) overall survival and (B) adverse event.

Funnel plots were generated regarding respective parametric data to detect relative publication bias. The results determined that no obvious publication bias was detected based on funnel-plots symmetries (Supplementary Figure S2-S7). By observing the network connections regarding different parametric data, we noticed that 2 closed loops existed in relative data namely, OS (Figure 2A) and AE (Figure 4A). Therefore, we conducted node-splitting analysis to detect any significant data inconsistency exist in the main results. The results demonstrated that no data inconsistency was found in our study with all *P* > 0.05 (Figure 5A- B). Meanwhile, all the calculations of pooled estimations were finished until PSRF became to 1. Thus, we concluded that our main results exhibited great data consistency and convergency.

## Discussion

In the current study, we included 31 RCTs investigating different targeted anticancer agents. With the samples size of 13023 participants with or without previous systematic treatments, 20 agents or combinations were reported for network pooled estimations. Finally, our results demonstrated lenvatinib and apatinib had superior effects on enhancing overall and progression-free survival, respectively, among the patients without previous systematic therapies. Meanwhile, regorafenib exhibited better efficacy on increasing overall and progression-free survival for patients administered with previous targeted agents. In addition, apatinib and codrituzumab had superior effects on reducing adverse event for participants with or without previous systematic treatments, respectively.

Lenvatinib is a novel first-line targeted anticancer agent for advanced HCC 25- 26. It is a multikinase inhibitor that targets VEGF receptors 1- 3, FGF receptors 1- 4, PDGF receptor α, RET, and KIT, which makes it reveal superior antitumour effects on multiple pathological mechanisms 27- 29. It was first approved in radioiodine-refractory differentiated thyroid cancer at a dose of 24 mg once daily 30. For HCC, lenvatinib was proved effective in some cohort studies with safety doses 31- 32, and it was the first anticancer agent that exhibited partial superior clinical effects than sorafenib 26. On the other hand, apatinib is a new type of small molecule tyrosine kinase inhibitor that mainly acts on VEGFR-2 33. This anti-angiogenesis targeted agent can normalize tumour angiogenesis before the vessel fading by improving the blood density, expansion and seepage, and enhance the penetration of chemotherapeutic agents and oxygen supply in a short time to increase the sensitivity to chemoradiation 34. To date, several clinical studies have determined that apatinib had clinical benefits for patients with advanced HCC. It may significantly prolong overall and progression- free survival 14, 35- 37. Apatinib could also provide clinical efficacy on gastric cancer and non-small cell lung cancer, illustrating its extensive oncological adaptability 38. More importantly, apatinib is a highly selective molecular inhibitor, and this may bring less cellular disorders with safety dose 39. These facts suggest that lenvatinib and apatinib have potentially superior clinical efficacy and safety for advanced HCC patient as primary systematic therapeutic agents. For patients who previously received targeted agents, regorafenib showed superior clinical efficacy on both overall and progression-free survival in the current study. Regorafenib is established as an oral multikinase inhibitor that targets vascular endothelial growth factor receptors1-3, platelet-derived growth factor receptor, fibroblast growth factor receptor, and colony-stimulating factor 1 receptor 40- 41. It had a distinct molecular target profile and had more potent pharmacological activity than sorafenib in preclinical studies 13. Prior to HCC, regorafenib was approved as monotherapy for the treatment of treatment-refractory metastatic colorectal cancer and gastrointestinal stromal tumours 42- 43. These features suggest that regorafenib may have better antitumour activity with broader anticancer epidemic and could replace sorafenib if no effective sorafenib benefit is detected. Moreover, this speculation was also demonstrated in clinical studies 16, 44, which may explain why regorafenib had superior effects on prolonging survival for patients with previously systematic treatments, especially for sorafenib- resistance or -intolerance.

Before the current study, 2 previous meta- analyses assessed 5-7 different targeted agents for advanced HCC 45- 46. These 2 publications made great works with appropriate procedures. However, many relative authoritative publications were not included in their analysis, and both of these papers only discussed previous agents as the first attempt. More importantly, these 2 previous reviews neglected to make an evaluation regarding with or without previous systematic treatment. A recent meta-analysis comparing several second-line agents made partial consistency conclusions as ours 47. Nevertheless, its lower inclusion criteria brought inevitable confounding factors, and they did not discuss the status and potential of second-line drugs further. More importantly, they ignored the relationships between so-called first- and second-line agents and failed to discuss future research directions. Based on these facts, we performed this comprehensive quantitative analysis and revised their conclusions. Our current study was the first network meta-analysis with further discussion which evaluated advanced HCC patients both with and without previously systematic treatments. According to our results, lenvatinib and apatinib had superior clinical efficacy and safety for patients without previously targeted therapies. These results were based on different probabilities; thus, direct statistical evidence was still inadequate. For example, lenvatinib had partially superior effects compared to sorafenib^26^. However, for apatinib, there was only 1 small sample retrospective investigation comparing apatinib and sorafenib which showed sorafenib was superior to apatinib 48. Moreover, apatinib administration was often combined with transarterial chemoembolization (TACE), and evaluation about adverse events may be influenced by other conditions. Nonetheless, we found the superior safety in of apatinib use in the current study. On the other hand, for patients who received previous systematic treatments, regorafenib had superior benefits but showed no advantage in lowering adverse event, unlike codrituzumab. However, tracing the raw data of included trials, 2 trials reporting on regorafenib and codrituzumab showed both of them had similar safety to placebo regarding adverse event in respective trails 16, 49. This may imply that regorafenib essentially bring similar safety compared to codrituzumab. Additionally, many so-called second-line agents were reported as the preparations for first-line agent failure or intolerance. Whether they have potential superior effects as first-line treatment remains unknown 50. Therefore, based on all these facts, we need more high-quality clinical trials to perfect our conclusions and these aforementioned issues should be the research directions in the future.

Although we finished a comprehensive quantitative analysis, we have to admit some inevitable drawbacks exist in our study. First, although we included 31 RCTs containing 13023 samples and no data inconsistency or bias were detected, many agents were reported from only 1 RCT. This may bring undetected bias to our conclusions. In addition, as mentioned above, our results were based on Bayesian theorem calculations and many issues lack direct statistical evidence. Due to lacking of relative RCTs, pair-wised could not be conducted, thus we draw our conclusions with caution and expected more trials in the future. Additionally, sensitivity analysis could not be performed and some confounding factors may be introduced into our analysis. Lastly, according to our high standards for inclusion criteria, we may have overlooked some meaningful literature. For these reasons, these results require further statistical validation and should be interpreted with caution.

In general, the current study finished a comprehensive evaluation of different targeted agent for advanced HCC patients with or without previously treatments. Based on the current evidence, we demonstrated that lenvatinib and apatinib may potentially have superior clinical efficacy for patients without previous targeted therapies, and regorafenib seemed to be the superior anticancer agent for patients with previous systematic treatments. On the other hand, our conclusions still need further statistical validations, and we are expecting more high-quality trials in the future.

## Supplementary Material

Supplementary figures and tables.

## Abbreviations

HCC: hepatocellular carcinoma
RCT: randomized controlled trial
OS: overall survival
PFS: progression-free surviva
AE: adverse event
PSRF: Potential Scale Reduction Factor
SUCRA: surface under the cumulative ranking
HBV: hepatitis B virus
HCV: hepatitis C virus
VEGF: vascular endothelial growth factor
PDGF: platelet-derived growth factor
PRISMA: Preferred Reporting Items for Systematic Reviews and Meta-Analyses
