# Supplementary material for: Evaluation of Targeted Agents for Advanced and Unresectable Hepatocellular Carcinoma: A Network Meta-Analysis
Source: J Cancer. 2019 Aug 19;10(19):4671–8. doi: 10.7150/jca.32828 (PMC6746130; doi:10.7150/jca.32828)

Supplementary Tables and Figures

Table S1. Example search strategy and process in MEDLINE.

# No.	Search	Results
1	exp (Hepatic) OR liver cancer/ti.ab	1131665
2	exp (Cancer) OR tumor/ti.ab	4163739
3	1 AND 2	305566
4	exp (Hepatocellular carcinoma) OR HCC/ti.ab	106515
5	3 OR 4	86841
6	exp ((advanced) OR unresectable) OR inoperable/ti.ab	535857
7	exp ((((((((((((((((Sorafenib) OR Codrituzumab) OR Cabozantinib) OR Regorafenib) OR Linifanib) OR Sunitinib) OR Tigatuzumab) OR Dovitinib) OR Mapatumumab) OR Vandetanib) OR Brivanib) OR Axitinib) OR Ramucirumab) OR Orantinib) OR Lenvatinib) OR Apatinib) OR Tivantinib) OR Bevacizumab) OR Erlotinib /ti.ab	34334
8	(random or prospective). ti,ab.	1186566
9	(randomized controlled trial). ti,ab.	611539
10	8 OR 9	145241
11	5 OR 6 OR 7	638059
12	11 AND 10	6977
13	human.sh.	18097130
14	animal.sh.	6486308
15	13 NOT 14	15905628
16	12 NOT 15	5884

Table S2. General information about the included RCTs. [OS, Overall Survival; PFS, Progression-Free Survival; AE, Adverse Event]

Author	Year	Region	Study arm	Sample size	Intervwntion	Administration	Previously systematic treated	Available data
Aboualfa	2010	USA	2	96	Sorafenib vs. NC	400 mg twice daily	No	OS; PFS; AE
Aboualfa	2016	USA	2	185	Codrituzumab vs. NC	1600 mg biweekly	Yes	OS; AE
Aboualfa	2018	USA	2	707	Cabozantinib vs. NC	60 mg once daily	Yes	OS; PFS; AE
Bruix	2015	Spain	2	1114	Sorafenib vs. NC	400 mg twice daily	No	OS; PFS; AE
Bruix	2017	Spain	2	573	Regorafenib vs. NC	160 mg once daily	Yes	OS; PFS; AE
Cainap	2015	Romania	2	1035	Linifanib vs. Sorafenib	17.5 mg once daily vs. 400 mg twice daily	No	OS; PFS; AE
Cheng	2009	Taiwan	2	226	Sorafenib vs. NC	400 mg twice daily	No	OS; PFS; AE
Cheng	2013	Taiwan	2	1074	Sunitinib vs. Sorafenib	37.5 mg once daily vs. 400 mg twice daily	No	OS; PFS; AE
Cheng	2015	Taiwan	2	162	Tigatuzumab + Sorafenib vs. Sorafenib	2 mg/kg/week maintenance + 400 mg twice daily vs. 400 mg twice daily	No	OS; PFS; AE
Cheng	2016	Taiwan	2	165	Dovitinib vs. Sorafenib	500 mg for 5 days per week vs. 400 mg twice daily	No	OS; PFS; AE
Ciuleanu	2016	Romania	2	101	Mapatumumab + Sorafenib vs. Sorafenib	30 mg/kg on Day 1 per 21 day cycle + 400 mg twice daily vs. 400 mg twice daily	No	OS; PFS; AE
Hsu	2012	Taiwan	2	67	Vandetanib vs. NC	300 or 100 mg once daily	No	OS; AE
Johnson	2013	USA	2	1155	Brivanib vs. Sorafenib	800 mg once daily vs. 400 mg twice daily	No	OS; PFS; AE
KAN	2015	China	2	62	Sorafenib vs. NC	400 mg twice daily	No	OS
Kang	2015	Korea	2	202	Axitinib vs. NC	5 mg twice daily	Yes	OS; PFS; AE
Kudo	2011	Japan	2	458	Sorafenib vs. NC	400 mg twice daily	No	OS; PFS; AE
Kudo	2014	Japan	2	502	Brivanib vs. NC	800 mg once daily	No	OS; AE
Kudo	2016	Japan	2	93	Ramucirumab vs. NC	8 mg/kg biweekly	Yes	OS; PFS; AE
Kudo	2017	Japan	2	888	Orantinib vs. NC	200 mg twice daily	No	OS; PFS; AE
Kudo	2018	Japan	2	954	Lenvatinib vs. Sorafenib	12 mg or 8 mg/day vs. 400 mg twice daily	No	OS; PFS; AE
Lencioni	2016	USA	2	307	Sorafenib vs. NC	400 mg twice daily	No	OS; PFS; AE
Llovet	2008	Spain	2	602	Sorafenib vs. NC	400 mg twice daily	No	OS; PFS; AE
Llovet	2013	Spain	2	395	Brivanib vs. NC	800 mg once daily	Yes	OS; PFS; AE
Lu	2017	China	2	42	Apatinib vs. NC	500 or 250 mg once daily	No	PFS; AE
Meyer	2017	UK	2	313	Sorafenib vs. NC	400 mg twice daily	No	OS; PFS; AE
Rimassa	2013	Italy	2	101	Sorafenib vs. NC	600 mg twice daily	Yes	OS
Sansonno	2012	Italy	2	62	Sorafenib vs. NC	400 mg twice daily	No	PFS; AE
Santoro	2013	Belgium	2	107	Tivantinib vs. NC	360 mg or 240 mg twice daily	Yes	OS; PFS; AE
Thomas	2018	USA	2	90	Bevacizumab + Erlotinib vs. Sorafenib	10 mg/kg Bevacizumab intravenously every 14 days + 150 mg Erlotinib orally daily vs. 400 mg twice daily	No	OS; PFS; AE
Zhu	2014	USA	2	720	Sorafenib + Erlotinib vs. Sorafenib	400 mg twice daily and 150 mg once daily for Erlotinib	No	OS; PFS; AE
Zhu	2015	USA	2	465	Ramucirumab vs. NC	8 mg/kg biweekly	Yes	OS; PFS; AE

References

1. Abou-Alfa GK, Johnson P, Knox JJ, Capanu M, Davidenko I, Lacava J, et al. Doxorubicin plus sorafenib vs doxorubicin alone in patients with advanced hepatocellular carcinoma: a randomized trial. JAMA. 2010; 304 (19): 2154- 60.

2. Abou-Alfa GK, Puig O, Daniele B, Kudo M, Merle P, Park JW, et al. Randomized phase II placebo controlled study of codrituzumab in previously treated patients with advanced hepatocellular carcinoma. J Hepatol. 2016; 65(2): 289- 95.

3. Abou-Alfa GK, Meyer T, Cheng AL, El-Khoueiry AB, Rimassa L, Ryoo BY, et al. Cabozantinib in Patients with Advanced and Progressing Hepatocellular Carcinoma. N Engl J Med. 2018; 379(1): 54- 63.

4. Bruix J, Takayama T, Mazzaferro V, Chau GY, Yang J, Kudo M, et al. Adjuvant sorafenib for hepatocellular carcinoma after resection or ablation (STORM): a phase 3, randomised, double-blind, placebo-controlled trial. Lancet Oncol. 2015; 16(13): 1344- 54.

5. Bruix J, Qin S, Merle P, Granito A, Huang YH, Bodoky G, et al. Regorafenib for patients with hepatocellular carcinoma who progressed on sorafenib treatment (RESORCE): a randomised, double-blind, placebo-controlled, phase 3 trial. Lancet. 2017; 389(10064): 56- 66.

6. Cainap C, Qin S, Huang WT, Chung IJ, Pan H, Cheng Y, et al. Linifanib versus Sorafenib in patients with advanced hepatocellular carcinoma: results of a randomized phase III trial. J Clin Oncol. 2015; 33(2): 172- 9.

7. Cheng AL, Kang YK, Chen Z, Tsao CJ, Qin S, Kim JS, et al. Efficacy and safety of sorafenib in patients in the Asia-Pacific region with advanced hepatocellular carcinoma: a phase III randomised, double-blind, placebo-controlled trial. Lancet Oncol. 2009; 10(1): 25- 34.

8. Cheng AL, Kang YK, Lin DY, Park JW, Kudo M, Qin S, et al. Sunitinib versus sorafenib in advanced hepatocellular cancer: results of a randomized phase III trial. J Clin Oncol. 2013; 31(32): 4067- 75.

9. Cheng AL, Kang YK, He AR, Lim HY, Ryoo BY, Hung CH, et al. Safety and efficacy of tigatuzumab plus sorafenib as first-line therapy in subjects with advanced hepatocellular carcinoma: A phase 2 randomized study. J Hepatol. 2015; 63(4): 896- 904.

10. Cheng AL, Thongprasert S, Lim HY, Sukeepaisarnjaroen W, Yang TS, Wu CC, et al. Randomized, open-label phase 2 study comparing frontline dovitinib versus sorafenib in patients with advanced hepatocellular carcinoma. Hepatology. 2016; 64(3): 774- 84.

11. Ciuleanu T, Bazin I, Lungulescu D, Miron L, Bondarenko I, Deptala A, et al. A randomized, double-blind, placebo-controlled phase II study to assess the efficacy and safety of mapatumumab with sorafenib in patients with advanced hepatocellular carcinoma. Ann Oncol. 2016; 27(4): 680- 7.

12. Hsu C, Yang TS, Huo TI, Hsieh RK, Yu CW, Hwang WS, et al. Vandetanib in patients with inoperable hepatocellular carcinoma: a phase II, randomized, double-blind, placebo-controlled study. J Hepatol. 2012; 56(5): 1097- 103.

13. Johnson PJ, Qin S, Park JW, Poon RT, Raoul JL, Philip PA, et al. Brivanib versus sorafenib as first-line therapy in patients with unresectable, advanced hepatocellular carcinoma: results from the randomized phase III BRISK-FL study. J Clin Oncol. 2013;31(28): 3517- 24.

14. Kan X, Jing Y, Wan QY, Pan JC, Han M, Yang Y, et al. Sorafenib combined with percutaneous radiofrequency ablation for the treatment of medium-sized hepatocellular carcinoma. Eur Rev Med Pharmacol Sci. 2015; 19(2): 247- 55.

15. Kang YK, Yau T, Park JW, Lim HY, Lee TY, Obi S, et al. Randomized phase II study of axitinib versus placebo plus best supportive care in second-line treatment of advanced hepatocellular carcinoma. Ann Oncol. 2015; 26(12): 2457- 63.

16. Kudo M, Imanaka K, Chida N, Nakachi K, Tak WY, Takayama T, et al. Phase III study of sorafenib after transarterial chemoembolisation in Japanese and Korean patients with unresectable hepatocellular carcinoma. Eur J Cancer. 2011; 47(14): 2117- 27.

17. Kudo M, Han G, Finn RS, Poon RT, Blanc JF, Yan L, et al. Brivanib as adjuvant therapy to transarterial chemoembolization in patients with hepatocellular carcinoma: A randomized phase III trial. Hepatology. 2014; 60(5): 1697- 707.

18. Kudo M, Hatano E, Ohkawa S, Fujii H, Masumoto A, Furuse J, et al. Ramucirumab as second-line treatment in patients with advanced hepatocellular carcinoma: Japanese subgroup analysis of the REACH trial. J Gastroenterol. 2017; 52(4): 494- 503.

19. Kudo M, Cheng AL, Park JW, Park JH, Liang PC, Hidaka H, et al. Orantinib versus placebo combined with transcatheter arterial chemoembolisation in patients with unresectable hepatocellular carcinoma (ORIENTAL): a randomised, double-blind, placebo-controlled, multicentre, phase 3 study. Lancet Gastroenterol Hepatol. 2018; 3(1): 37- 46.

20. Kudo M, Finn RS, Qin S, Han KH, Ikeda K, Piscaglia F, et al. Lenvatinib versus sorafenib in first-line treatment of patients with unresectable hepatocellular carcinoma: a randomised phase 3 non-inferiority trial. Lancet. 2018; 391(10126): 1163- 1173.

21. Lencioni R, Llovet JM, Han G, Tak WY, Yang J, Guglielmi A, et al. Sorafenib or placebo plus TACE with doxorubicin-eluting beads for intermediate stage HCC: The SPACE trial. J Hepatol. 2016; 64(5): 1090- 1098.

22. Llovet JM, Ricci S, Mazzaferro V, Hilgard P, Gane E, Blanc JF, et al. Sorafenib in advanced hepatocellular carcinoma. N Engl J Med. 2008; 359(4): 378- 90.

23. Llovet JM, Decaens T, Raoul JL, Boucher E, Kudo M, Chang C, et al. Brivanib in patients with advanced hepatocellular carcinoma who were intolerant to sorafenib or for whom sorafenib failed: results from the randomized phase III BRISK-PS study. J Clin Oncol. 2013; 31(28): 3509- 16.

24. Lu W, Jin XL, Yang C, Du P, Jiang FQ, Ma JP, et al. Comparison of efficacy between TACE combined with apatinib and TACE alone in the treatment of intermediate and advanced hepatocellular carcinoma: A single-center randomized controlled trial. Cancer Biol Ther. 2017; 18(6): 433- 438.

25. Meyer T, Fox R, Ma YT, Ross PJ, James MW, Sturgess R, et al. Sorafenib in combination with transarterial chemoembolisation in patients with unresectable hepatocellular carcinoma (TACE 2): a randomised placebo-controlled, double-blind, phase 3 trial. Lancet Gastroenterol Hepatol. 2017; 2(8): 565- 575.

26. Rimassa L, Pressiani T, Boni C, Carnaghi C, Rota Caremoli E, et al. A phase II randomized dose escalation trial of sorafenib in patients with advanced hepatocellular carcinoma. Oncologist. 2013; 18(4): 379- 80.

27. Sansonno D, Lauletta G, Russi S, Conteduca V, Sansonno L, Dammacco F. Transarterial chemoembolization plus sorafenib: a sequential therapeutic scheme for HCV-related intermediate-stage hepatocellular carcinoma: a randomized clinical trial. Oncologist. 2012; 17(3): 359- 66.

28. Santoro A, Rimassa L, Borbath I, Daniele B, Salvagni S, Van Laethem JL, et al. Tivantinib for second-line treatment of advanced hepatocellular carcinoma: a randomised, placebo-controlled phase 2 study. Lancet Oncol. 2013; 14(1): 55- 63.

29. Thomas MB, Garrett-Mayer E, Anis M, Anderton K, Bentz T, Edwards A, et al. A Randomized Phase II Open-Label Multi-Institution Study of the Combination of Bevacizumab and Erlotinib Compared to Sorafenib in the First-Line Treatment of Patients with Advanced Hepatocellular Carcinoma. Oncology. 2018; 94(6): 329- 339.

30. Zhu AX, Rosmorduc O, Evans TR, Ross PJ, Santoro A, Carrilho FJ, et al. SEARCH: a phase III, randomized, double-blind, placebo-controlled trial of sorafenib plus erlotinib in patients with advanced hepatocellular carcinoma. J Clin Oncol. 2015; 33(6): 559- 66.

31. Zhu AX, Park JO, Ryoo BY, Yen CJ, Poon R, Pastorelli D, et al. Ramucirumab versus placebo as second-line treatment in patients with advanced hepatocellular carcinoma following first-line therapy with sorafenib (REACH): a randomised, double-blind, multicentre, phase 3 trial. Lancet Oncol. 2015; 16(7): 859- 70.

Table S3. Comparisons of included agents in network meta-analysis regarding overall survival. Odds ratios are presented in the cells in common between the column-defining and row-defining agents. [OR: odds ratio; CI: credible intervals].

Item	Comparison [OR (95%CI)]												
No previously systematic treated	Bevacizumab + Erlotinib	1.89 (0.44, 7.76)	0.89 (0.16, 4.98)	2.29 (0.49, 11.01)	1.70 (0.35, 8.44)	1.68 (0.30, 9.23)	1.84 (0.47, 6.74)	1.70 (0.31, 8.26)	1.89 (0.53, 6.65)	1.92 (0.40, 9.10)	1.50 (0.31, 7.16)	1.68 (0.30, 8.84)	1.26 (0.15, 10.58)
	0.53 (0.13, 2.26)	Brivanib	0.47 (0.12, 1.89)	1.21 (0.37, 4.17)	0.89 (0.28, 3.11)	0.88 (0.22, 3.66)	0.98 (0.47, 1.97)	0.90 (0.25, 2.88)	1.00 (0.50, 2.09)	1.01 (0.32, 3.54)	0.79 (0.24, 2.64)	0.88 (0.23, 3.51)	0.67 (0.11, 4.00)
	1.12 (0.20, 6.30)	2.11 (0.53, 8.33)	Dovitinib	2.57 (0.58, 11.54)	1.90 (0.42, 8.76)	1.88 (0.34, 10.11)	2.07 (0.61, 6.77)	1.90 (0.38, 8.52)	2.13 (0.67, 6.79)	2.13 (0.49, 9.99)	1.67 (0.38, 7.18)	1.86 (0.36, 9.73)	1.43 (0.18, 10.98)
	0.44 (0.09, 2.05)	0.82 (0.24, 2.73)	0.39 (0.09, 1.73)	Lenvatinib	0.75 (0.19, 2.97)	0.72 (0.16, 3.37)	0.81 (0.27, 2.21)	0.74 (0.16, 2.99)	0.82 (0.31, 2.18)	0.84 (0.21, 3.23)	0.65 (0.17, 2.37)	0.73 (0.16, 3.12)	0.56 (0.08, 3.84)
	0.59 (0.12, 2.86)	1.12 (0.32, 3.61)	0.53 (0.11, 2.38)	1.34 (0.34, 5.22)	Linifanib	0.99 (0.21, 4.53)	1.10 (0.37, 3.02)	1.00 (0.23, 3.88)	1.12 (0.42, 2.92)	1.14 (0.28, 4.40)	0.88 (0.22, 3.44)	0.99 (0.21, 4.28)	0.75 (0.11, 5.26)
	0.60 (0.11, 3.28)	1.13 (0.27, 4.51)	0.53 (0.10, 2.92)	1.38 (0.30, 6.28)	1.01 (0.22, 4.67)	Mapatumumab + Sorafenib	1.11 (0.30, 3.83)	1.01 (0.19, 4.76)	1.13 (0.33, 3.78)	1.16 (0.24, 5.27)	0.89 (0.19, 4.14)	0.99 (0.20, 5.24)	0.75 (0.09, 6.21)
	0.54 (0.15, 2.11)	1.02 (0.51, 2.13)	0.48 (0.15, 1.64)	1.23 (0.45, 3.76)	0.91 (0.33, 2.72)	0.90 (0.26, 3.36)	NC	0.91 (0.33, 2.35)	1.02 (0.71, 1.56)	1.03 (0.39, 3.16)	0.80 (0.30, 2.36)	0.90 (0.27, 3.09)	0.68 (0.13, 3.68)
	0.59 (0.12, 3.27)	1.11 (0.35, 3.95)	0.53 (0.12, 2.64)	1.35 (0.33, 6.26)	1.00 (0.26, 4.43)	0.99 (0.21, 5.18)	1.09 (0.42, 3.02)	Orantinib	1.11 (0.42, 3.48)	1.13 (0.29, 5.33)	0.88 (0.22, 3.91)	1.00 (0.21, 4.81)	0.75 (0.12, 5.19)
	0.53 (0.15, 1.88)	1.00 (0.48, 2.01)	0.47 (0.15, 1.48)	1.21 (0.46, 3.23)	0.89 (0.34, 2.38)	0.88 (0.26, 3.01)	0.98 (0.64, 1.40)	0.90 (0.29, 2.38)	Sorafenib	1.01 (0.39, 2.76)	0.79 (0.29, 2.05)	0.88 (0.28, 2.77)	0.66 (0.12, 3.70)
	0.52 (0.11, 2.50)	0.99 (0.28, 3.16)	0.47 (0.10, 2.05)	1.19 (0.31, 4.68)	0.88 (0.23, 3.56)	0.86 (0.19, 4.13)	0.97 (0.32, 2.57)	0.89 (0.19, 3.44)	0.99 (0.36, 2.59)	Sorafenib + Erlotinib	0.78 (0.19, 3.10)	0.87 (0.20, 3.80)	0.66 (0.09, 4.67)

	0.66 (0.14, 3.24)	1.27 (0.38, 4.23)	0.60 (0.14, 2.65)	1.54 (0.42, 5.97)	1.14 (0.29, 4.53)	1.12 (0.24, 5.32)	1.25 (0.42, 3.39)	1.14 (0.26, 4.45)	1.27 (0.49, 3.40)	1.29 (0.32, 5.13)	Sunitinib	1.12 (0.26, 4.96)	0.84 (0.12, 5.96)
	0.60 (0.11, 3.33)	1.13 (0.28, 4.33)	0.54 (0.10, 2.76)	1.37 (0.32, 6.24)	1.01 (0.23, 4.68)	1.01 (0.19, 5.13)	1.12 (0.32, 3.66)	1.00 (0.21, 4.69)	1.13 (0.36, 3.58)	1.14 (0.26, 5.10)	0.89 (0.20, 3.87)	Tigatuzumab + Sorafenib	0.76 (0.10, 5.88)
	0.79 (0.09, 6.53)	1.49 (0.25, 8.75)	0.70 (0.09, 5.61)	1.80 (0.26, 12.64)	1.34 (0.19, 9.14)	1.33 (0.16, 11.28)	1.47 (0.27, 7.42)	1.34 (0.19, 8.56)	1.51 (0.27, 8.34)	1.52 (0.21, 11.15)	1.19 (0.17, 8.27)	1.32 (0.17, 10.32)	Vandetanib
**Previously systematic treated**	Axitinib	0.66 (0.12, 3.34)	0.91 (0.18, 4.70)	0.77 (0.13, 4.41)	0.57 (0.17, 1.82)	0.91 (0.21, 3.97)	1.43 (0.27, 7.50)	0.74 (0.10, 5.09)	0.69 (0.11, 4.25)				
	1.52 (0.30, 8.25)	Brivanib	1.39 (0.30, 7.26)	1.18 (0.22, 6.57)	0.87 (0.28, 2.82)	1.38 (0.35, 6.16)	2.21 (0.46, 11.04)	1.13 (0.17, 8.02)	1.07 (0.17, 6.44)				
	1.10 (0.21, 5.64)	0.72 (0.14, 3.33)	Cabozantinib	0.86 (0.15, 4.59)	0.62 (0.20, 1.88)	0.99 (0.26, 4.34)	1.59 (0.32, 7.54)	0.80 (0.12, 5.41)	0.76 (0.13, 4.38)				
	1.30 (0.23, 7.83)	0.85 (0.15, 4.60)	1.16 (0.22, 6.48)	Codrituzumab	0.74 (0.20, 2.73)	1.17 (0.26, 6.02)	1.83 (0.34, 10.69)	0.95 (0.13, 7.51)	0.89 (0.13, 5.76)				
	1.75 (0.55, 5.96)	1.15 (0.35, 3.55)	1.61 (0.53, 4.92)	1.35 (0.37, 5.02)	NC	1.59 (0.71, 4.08)	2.54 (0.83, 7.98)	1.29 (0.27, 6.26)	1.22 (0.30, 4.78)				
	1.10 (0.25, 4.73)	0.73 (0.16, 2.85)	1.01 (0.23, 3.82)	0.86 (0.17, 3.87)	0.63 (0.25, 1.41)	Ramucirumab	1.61 (0.36, 6.22)	0.79 (0.13, 4.57)	0.77 (0.14, 3.60)				
	0.70 (0.13, 3.68)	0.45 (0.09, 2.17)	0.63 (0.13, 3.17)	0.55 (0.09, 2.98)	0.39 (0.13, 1.20)	0.62 (0.16, 2.77)	Regorafenib	0.51 (0.07, 3.50)	0.49 (0.08, 2.70)				
	1.34 (0.20, 9.85)	0.88 (0.12, 5.88)	1.26 (0.18, 8.17)	1.05 (0.13, 7.93)	0.77 (0.16, 3.65)	1.27 (0.22, 7.80)	1.97 (0.29, 13.45)	Sorafenib	0.94 (0.12, 7.11)				
	1.44 (0.24, 9.37)	0.93 (0.16, 5.72)	1.31 (0.23, 7.82)	1.13 (0.17, 7.86)	0.82 (0.21, 3.32)	1.30 (0.28, 7.15)	2.06 (0.37, 12.89)	1.07 (0.14, 8.51)	Tivantinib				

Table S4. Comparisons of included agents in network meta-analysis regarding progression-free survival. Odds ratios are presented in the cells in common between the column-defining and row-defining agents. [OR: odds ratio; CI: credible intervals].

**Item**	**Comparison [OR (95%CI)]**												
**No previously systematic treated**	Apatinib	0.45 (0.02, 11.83)	0.22 (0.01, 4.32)	0.05 (0.00, 1.24)	0.72 (0.05, 13.29)	0.38 (0.03, 6.89)	0.34 (0.02, 7.90)	0.28 (0.03, 2.40)	0.51 (0.03, 8.29)	0.33 (0.04, 3.61)	0.35 (0.02, 6.61)	0.16 (0.01, 3.03)	0.30 (0.02, 6.23)
	2.21 (0.08, 40.65)	Bevacizumab + Erlotinib	0.47 (0.03, 6.90)	0.11 (0.00, 2.37)	1.58 (0.10, 25.72)	0.83 (0.05, 12.37)	0.74 (0.04, 13.37)	0.62 (0.06, 4.91)	1.11 (0.06, 16.74)	0.73 (0.09, 5.81)	0.78 (0.05, 12.54)	0.36 (0.02, 5.20)	0.66 (0.04, 10.43)
	4.61 (0.23, 71.75)	2.12 (0.14, 31.25)	Brivanib	0.23 (0.01, 4.04)	3.38 (0.25, 42.45)	1.75 (0.14, 22.44)	1.55 (0.12, 21.06)	1.34 (0.17, 7.64)	2.41 (0.15, 29.74)	1.54 (0.26, 9.32)	1.62 (0.13, 21.60)	0.75 (0.06, 9.37)	1.39 (0.11, 18.03)
	21.24 (0.81, 502.84)	9.32 (0.42, 266.15)	4.39 (0.25, 96.26)	Dovitinib	14.81 (0.83, 354.83)	7.96 (0.44, 155.74)	6.88 (0.36, 193.58)	5.81 (0.50, 75.63)	10.55 (0.48, 231.33)	6.91 (0.74, 87.42)	7.31 (0.44, 161.61)	3.39 (0.19, 69.31)	6.28 (0.36, 144.40)
	1.40 (0.08, 19.83)	0.63 (0.04, 9.87)	0.30 (0.02, 3.95)	0.07 (0.00, 1.21)	Lenvatinib	0.52 (0.04, 6.19)	0.47 (0.03, 6.55)	0.40 (0.05, 2.31)	0.72 (0.05, 8.56)	0.46 (0.08, 2.84)	0.49 (0.04, 6.30)	0.23 (0.02, 2.94)	0.42 (0.03, 5.50)
	2.64 (0.15, 38.27)	1.21 (0.08, 19.63)	0.57 (0.04, 7.18)	0.13 (0.01, 2.26)	1.91 (0.16, 22.23)	Linifanib	0.91 (0.06, 11.87)	0.76 (0.10, 4.40)	1.37 (0.08, 15.86)	0.89 (0.15, 5.04)	0.94 (0.08, 11.46)	0.43 (0.04, 5.46)	0.79 (0.07, 9.88)

	2.98 (0.13, 49.13)	1.36 (0.07, 23.57)	0.64 (0.05, 8.39)	0.15 (0.01, 2.80)	2.11 (0.15, 28.84)	1.10 (0.08, 15.56)	Mapatumumab + Sorafenib	0.84 (0.09, 5.76)	1.54 (0.08, 19.83)	0.98 (0.14, 6.43)	1.05 (0.08, 15.52)	0.48 (0.03, 6.43)	0.89 (0.06, 12.54)
	3.52 (0.42, 29.69)	1.62 (0.20, 16.87)	0.75 (0.13, 6.01)	0.17 (0.01, 2.02)	2.48 (0.43, 19.24)	1.31 (0.23, 9.77)	1.18 (0.17, 10.90)	NC	1.78 (0.31, 11.08)	1.16 (0.67, 2.75)	1.21 (0.20, 10.14)	0.57 (0.10, 4.59)	1.06 (0.19, 8.29)
	1.98 (0.12, 30.25)	0.90 (0.06, 17.71)	0.42 (0.03, 6.50)	0.09 (0.00, 2.06)	1.38 (0.12, 20.60)	0.73 (0.06, 11.94)	0.65 (0.05, 11.79)	0.56 (0.09, 3.27)	Orantinib	0.65 (0.11, 4.95)	0.68 (0.06, 11.60)	0.31 (0.03, 4.82)	0.58 (0.05, 9.52)
	3.01 (0.28, 24.52)	1.38 (0.17, 11.14)	0.65 (0.11, 3.79)	0.14 (0.01, 1.36)	2.18 (0.35, 12.10)	1.13 (0.20, 6.58)	1.02 (0.16, 7.19)	0.86 (0.36, 1.50)	1.54 (0.20, 8.94)	Sorafenib	1.05 (0.18, 6.45)	0.49 (0.08, 2.97)	0.91 (0.15, 5.38)
	2.89 (0.15, 41.38)	1.29 (0.08, 21.63)	0.62 (0.05, 7.45)	0.14 (0.01, 2.29)	2.04 (0.16, 25.32)	1.07 (0.09, 12.11)	0.95 (0.06, 13.09)	0.82 (0.10, 4.88)	1.48 (0.09, 17.41)	0.95 (0.15, 5.56)	Sorafenib + Erlotinib	0.47 (0.04, 5.73)	0.86 (0.07, 10.87)
	6.13 (0.33, 91.26)	2.81 (0.19, 45.44)	1.34 (0.11, 16.38)	0.29 (0.01, 5.25)	4.39 (0.34, 57.04)	2.31 (0.18, 28.12)	2.08 (0.16, 28.67)	1.77 (0.22, 10.20)	3.20 (0.21, 37.21)	2.02 (0.34, 12.42)	2.14 (0.17, 27.55)	Sunitinib	1.85 (0.15, 22.42)
	3.35 (0.16, 50.25)	1.51 (0.10, 23.92)	0.72 (0.06, 8.87)	0.16 (0.01, 2.76)	2.36 (0.18, 30.21)	1.26 (0.10, 14.65)	1.12 (0.08, 16.48)	0.95 (0.12, 5.37)	1.71 (0.11, 19.47)	1.10 (0.19, 6.59)	1.16 (0.09, 15.34)	0.54 (0.04, 6.62)	Tigatuzumab + Sorafenib
**Previously systematic treated**	Axitinib	0.75 (0.02, 53.24)	1.71 (0.07, 30.53)	0.38 (0.03, 3.11)	1.53 (0.08, 21.32)	2.03 (0.08, 42.13)	0.47 (0.01, 37.33)						
	1.34 (0.02, 57.22)	Brivanib	2.23 (0.04, 67.63)	0.51 (0.01, 7.33)	1.98 (0.04, 47.59)	2.87 (0.05, 84.25)	0.60 (0.01, 54.12)						
	0.59 (0.03, 13.81)	0.45 (0.01, 25.66)	Cabozantinib	0.22 (0.03, 1.38)	0.87 (0.08, 10.87)	1.20 (0.07, 21.79)	0.28 (0.01, 18.42)						
	2.65 (0.32, 34.54)	1.96 (0.14, 79.48)	4.50 (0.73, 33.94)	NC	3.98 (1.03, 19.48)	5.36 (0.73, 47.10)	1.25 (0.06, 55.22)						
	0.66 (0.05, 12.41)	0.51 (0.02, 24.79)	1.15 (0.09, 12.81)	0.25 (0.05, 0.97)	Ramucirumab	1.31 (0.11, 17.79)	0.32 (0.01, 17.77)						
	0.49 (0.02, 12.31)	0.35 (0.01, 21.91)	0.83 (0.05, 13.50)	0.19 (0.02, 1.36)	0.76 (0.06, 9.52)	Regorafenib	0.25 (0.01, 16.59)						
	2.15 (0.03, 112.05)	1.67 (0.02, 137.33)	3.57 (0.05, 144.08)	0.80 (0.02, 15.84)	3.12 (0.06, 94.99)	4.03 (0.06, 166.39)	Tivantinib						

Table S5. Comparisons of included agents in network meta-analysis regarding adverse event. Odds ratios are presented in the cells in common between the column-defining and row-defining agents. [OR: odds ratio; CI: credible intervals].

**Item**	**Comparison [OR (95%CI)]**													
**No previously systematic treated**	Apatinib	3.17 (0.00, 32160.78)	2.05 (0.00, 3135.78)	3.08 (0.00, 29320.98)	2.45 (0.00, 14729.47)	5.29 (0.00, 27492.04)	4.05 (0.00, 18292.07)	0.16 (0.00, 65.73)	4.74 (0.00, 20046.78)	3.14 (0.00, 1646.65)	104.37 (0.02, 824292.71)	8.35 (0.00, 56262.14)	15.54 (0.00, 111796.03)	1.11 (0.00, 5014.77)
	0.32 (0.00, 3146.35)	Bevacizumab + Erlotinib	0.61 (0.00, 949.00)	0.96 (0.00, 7945.64)	0.74 (0.00, 3507.24)	1.55 (0.00, 6908.33)	1.18 (0.00, 4694.11)	0.05 (0.00, 35.70)	1.45 (0.00, 8796.60)	0.92 (0.00, 504.43)	31.79 (0.00, 215321.94)	2.53 (0.00, 16668.93)	4.68 (0.00, 34327.54)	0.34 (0.00, 2480.12)
	0.49 (0.00, 1007.52)	1.65 (0.00, 3358.57)	Brivanib	1.55 (0.00, 2708.73)	1.27 (0.00, 1306.37)	2.67 (0.00, 2987.94)	2.01 (0.00, 1806.81)	0.08 (0.00, 4.50)	2.33 (0.00, 2297.37)	1.57 (0.03, 90.42)	50.11 (0.04, 85533.05)	3.98 (0.00, 9289.51)	7.87 (0.01, 16957.41)	0.55 (0.00, 680.90)

	0.33 (0.00, 2855.09)	1.04 (0.00, 13218.94)	0.65 (0.00, 1137.20)	Dovitinib	0.80 (0.00, 4068.91)	1.69 (0.00, 7590.83)	1.33 (0.00, 7295.11)	0.05 (0.00, 44.46)	1.52 (0.00, 10773.57)	0.99 (0.00, 632.52)	34.46 (0.01, 248052.67)	2.64 (0.00, 25851.55)	5.17 (0.00, 50622.56)	0.36 (0.00, 2905.53)
	0.41 (0.00, 2076.89)	1.35 (0.00, 7588.24)	0.79 (0.00, 747.60)	1.24 (0.00, 5013.17)	Lenvatinib	2.08 (0.00, 6533.55)	1.57 (0.00, 4145.07)	0.06 (0.00, 21.95)	1.88 (0.00, 6873.15)	1.21 (0.00, 326.54)	41.21 (0.01, 157466.86)	3.44 (0.00, 16767.77)	6.36 (0.00, 33889.11)	0.44 (0.00, 1781.14)
	0.19 (0.00, 1126.83)	0.64 (0.00, 3680.00)	0.37 (0.00, 465.67)	0.59 (0.00, 2905.00)	0.48 (0.00, 1486.90)	Linifanib	0.76 (0.00, 2048.33)	0.03 (0.00, 10.62)	0.90 (0.00, 2976.51)	0.59 (0.00, 155.52)	18.35 (0.01, 73684.72)	1.59 (0.00, 8077.68)	3.10 (0.00, 13071.06)	0.21 (0.00, 1006.85)
	0.25 (0.00, 1380.72)	0.84 (0.00, 4763.01)	0.50 (0.00, 456.25)	0.75 (0.00, 3509.78)	0.64 (0.00, 1585.88)	1.31 (0.00, 3419.10)	Mapatumumab + Sorafenib	0.04 (0.00, 12.78)	1.16 (0.00, 2943.27)	0.77 (0.00, 199.36)	25.15 (0.01, 105339.38)	2.05 (0.00, 8824.09)	3.86 (0.00, 18997.82)	0.29 (0.00, 1059.09)
	6.12 (0.02, 3604.54)	20.76 (0.03, 22182.97)	12.26 (0.22, 766.87)	20.16 (0.02, 14386.30)	15.80 (0.05, 5704.41)	33.25 (0.09, 12922.95)	25.04 (0.08, 9201.74)	NC	29.62 (0.13, 8417.04)	19.27 (2.84, 139.11)	635.37 (1.28, 495560.47)	51.63 (0.08, 43539.98)	97.01 (0.19, 87734.34)	7.19 (0.02, 2201.76)
	0.21 (0.00, 933.57)	0.69 (0.00, 4752.12)	0.43 (0.00, 456.60)	0.66 (0.00, 3794.55)	0.53 (0.00, 2061.28)	1.12 (0.00, 3591.81)	0.86 (0.00, 2490.40)	0.03 (0.00, 7.52)	Orantinib	0.65 (0.00, 245.51)	19.96 (0.01, 109675.69)	1.84 (0.00, 8365.30)	3.12 (0.00, 20553.19)	0.24 (0.00, 801.14)
	0.32 (0.00, 234.41)	1.09 (0.00, 795.52)	0.64 (0.01, 38.35)	1.01 (0.00, 576.51)	0.82 (0.00, 240.45)	1.70 (0.01, 471.79)	1.29 (0.01, 334.56)	0.05 (0.01, 0.35)	1.54 (0.00, 525.49)	Sorafenib	31.96 (0.09, 17046.86)	2.65 (0.01, 1672.46)	5.08 (0.01, 3264.62)	0.37 (0.00, 156.45)
	0.01 (0.00, 63.04)	0.03 (0.00, 200.86)	0.02 (0.00, 26.03)	0.03 (0.00, 191.88)	0.02 (0.00, 94.47)	0.05 (0.00, 167.55)	0.04 (0.00, 118.00)	0.00 (0.00, 0.78)	0.05 (0.00, 192.45)	0.03 (0.00, 11.37)	Sorafenib + Erlotinib	0.08 (0.00, 537.38)	0.16 (0.00, 853.56)	0.01 (0.00, 58.59)
	0.12 (0.00, 760.37)	0.40 (0.00, 3137.81)	0.25 (0.00, 363.26)	0.38 (0.00, 2252.32)	0.29 (0.00, 1210.12)	0.63 (0.00, 3659.91)	0.49 (0.00, 1820.57)	0.02 (0.00, 12.91)	0.54 (0.00, 3386.19)	0.38 (0.00, 194.37)	12.13 (0.00, 75597.97)	Sunitinib	1.96 (0.00, 13014.64)	0.13 (0.00, 911.71)
	0.06 (0.00, 373.94)	0.21 (0.00, 1549.81)	0.13 (0.00, 174.35)	0.19 (0.00, 1101.66)	0.16 (0.00, 604.26)	0.32 (0.00, 1032.95)	0.26 (0.00, 731.09)	0.01 (0.00, 5.40)	0.32 (0.00, 1249.27)	0.20 (0.00, 80.63)	6.09 (0.00, 34563.38)	0.51 (0.00, 3412.68)	Tigatuzumab + Sorafenib	0.07 (0.00, 351.71)
	0.90 (0.00, 5460.05)	2.91 (0.00, 24780.69)	1.83 (0.00, 2103.26)	2.78 (0.00, 17456.47)	2.25 (0.00, 9446.52)	4.70 (0.00, 15810.74)	3.47 (0.00, 14677.23)	0.14 (0.00, 48.91)	4.24 (0.00, 14169.65)	2.72 (0.01, 1475.51)	92.59 (0.02, 489495.42)	7.45 (0.00, 51459.57)	14.18 (0.00, 110526.32)	Vandetanib
**Previously systematic treated**	Axitinib	0.20 (0.00, 23.54)	3.60 (0.02, 539.84)	0.03 (0.00, 6.32)	0.31 (0.01, 10.39)	0.63 (0.01, 71.90)	10.03 (0.06, 3806.62)	7.63 (0.03, 1706.58)						
	5.10 (0.04, 1106.26)	Brivanib	19.95 (0.15, 3408.99)	0.18 (0.00, 38.94)	1.55 (0.06, 56.74)	3.46 (0.07, 410.04)	59.84 (0.50, 20140.34)	36.72 (0.29, 8821.40)						
	0.28 (0.00, 56.10)	0.05 (0.00, 6.86)	Cabozantinib	0.01 (0.00, 2.06)	0.08 (0.00, 3.04)	0.20 (0.00, 22.63)	3.37 (0.02, 1283.53)	2.01 (0.01, 390.81)						
	29.20 (0.16, 10775.08)	5.69 (0.03, 1271.89)	117.22 (0.49, 27871.97)	Codrituzumab	8.50 (0.18, 732.30)	20.79 (0.24, 4791.14)	349.25 (1.36, 261932.69)	216.88 (0.99, 70997.61)						
	3.22 (0.10, 178.31)	0.64 (0.02, 17.89)	12.05 (0.33, 408.07)	0.12 (0.00, 5.71)	NC	2.22 (0.26, 42.81)	38.02 (1.01, 4086.62)	22.85 (0.60, 1180.17)						
	1.58 (0.01, 119.91)	0.29 (0.00, 13.35)	5.02 (0.04, 288.55)	0.05 (0.00, 4.23)	0.45 (0.02, 3.88)	Ramucirumab	14.54 (0.15, 2478.27)	11.31 (0.09, 880.15)						
	0.10 (0.00, 16.85)	0.02 (0.00, 2.01)	0.30 (0.00, 45.55)	0.00 (0.00, 0.74)	0.03 (0.00, 0.99)	0.07 (0.00, 6.75)	Regorafenib	0.71 (0.00, 146.95)						
	0.13 (0.00, 28.75)	0.03 (0.00, 3.44)	0.50 (0.00, 74.72)	0.00 (0.00, 1.02)	0.04 (0.00, 1.68)	0.09 (0.00, 10.61)	1.40 (0.01, 742.04)	Tivantinib						

Figure S1. Quality assessment of (A) overall and (B) study-level risk of bias, using Cochrane's risk of bias assessment tool, indicating quality with high ratings, unclear or low risk of bias, according to 6 evaluation criteria for each study.

Figure S1A

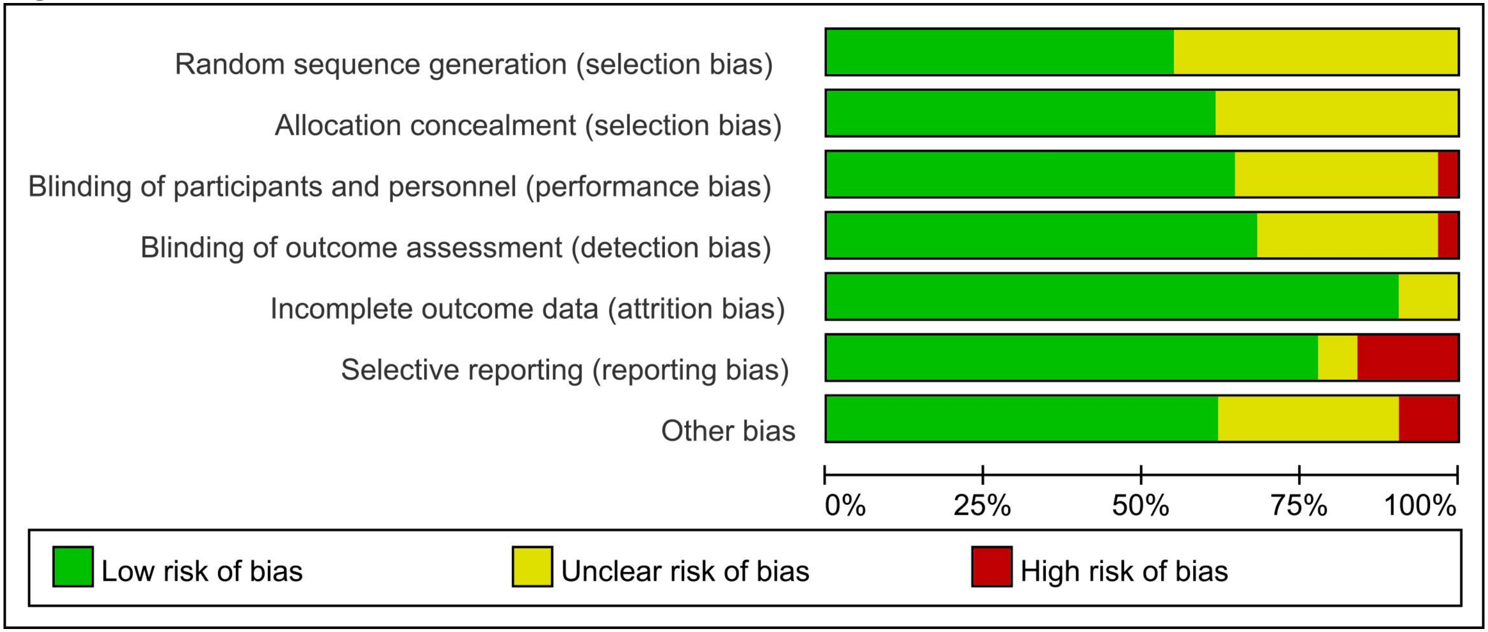

Figure S1B

	Random sequence generation (selection bias)	Allocation concealment (selection bias)	Blinding of participants and personnel (performance bias)	Blinding of outcome assessment (detection bias)	Incomplete outcome data (attrition bias)	Selective reporting (reporting bias)	Other bias
Aboualfa 2010	+	+	+	+	+	+	+
Aboualfa 2016	?	?	?	?	+	+	?
Aboualfa 2018	+	+	+	+	+	+	+
Bruix 2015	+	+	+	+	+	+	+
Bruix 2017	+	+	+	+	+	+	?
Cainap 2015	?	+	−	−	?	?	+
Cheng 2009	?	?	+	+	+	+	?
Cheng 2013	?	?	?	?	+	+	+
Cheng 2015	?	?	?	?	+	+	+
Cheng 2016	?	?	?	?	+	?	?
Ciuleanu 2016	?	?	+	+	+	+	+
Hsu 2012	+	+	+	+	+	+	+
Johnson 2013	+	+	+	+	+	+	+
KAN 2015	?	?	?	?	+	−	−
Kang 2015	+	+	+	+	+	+	+
Kudo 2011	?	?	+	+	+	−	?
Kudo 2014	+	+	+	+	+	+	+
Kudo 2016	+	+	+	+	+	+	+
Kudo 2017	+	+	+	+	+	+	+
Kudo 2018	+	+	?	?	+	+	+
Lencioni 2016	+	+	?	+	+	+	+
Llovet 2008	+	+	+	+	+	+	+
Llovet 2013	?	?	+	+	+	+	?
Lu 2017	?	?	?	?	+	−	?
Meyer 2017	+	+	+	+	+	+	+
Rimassa 2013	?	?	?	?	+	−	−
Sansonno 2012	?	+	+	+	+	+	?
Santoro 2013	+	+	+	+	?	−	−
Thomas 2018	?	?	?	?	?	+	?
Zhu 2014	+	+	+	+	+	+	+
Zhu 2015	+	+	+	+	+	+	+

Figure S2. Publication bias testing by funnel plot regarding overall survival of patients without previous systematic therapy.

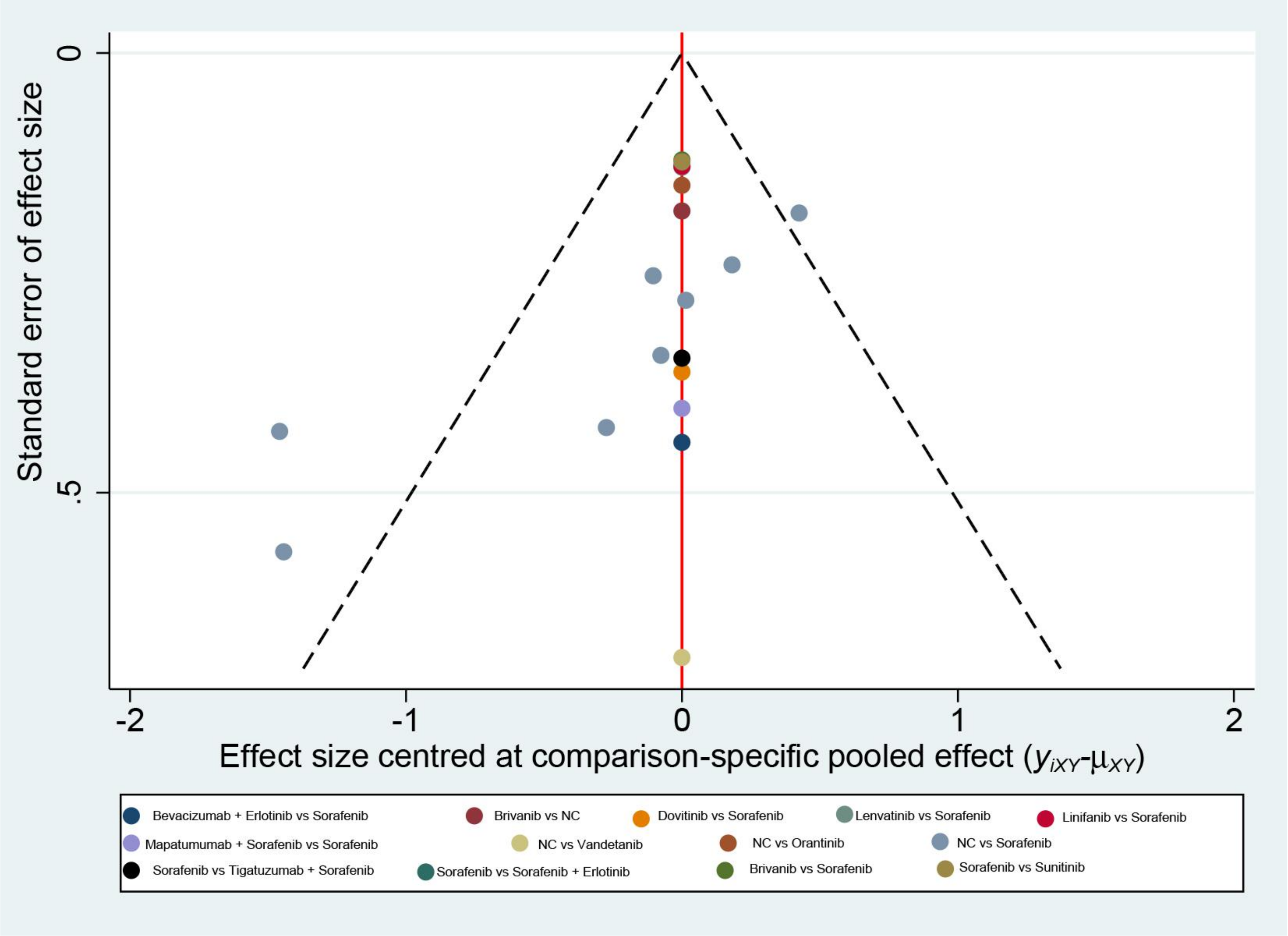

Figure S3. Publication bias testing by funnel plot regarding overall survival of patients with previous systematic therapy.

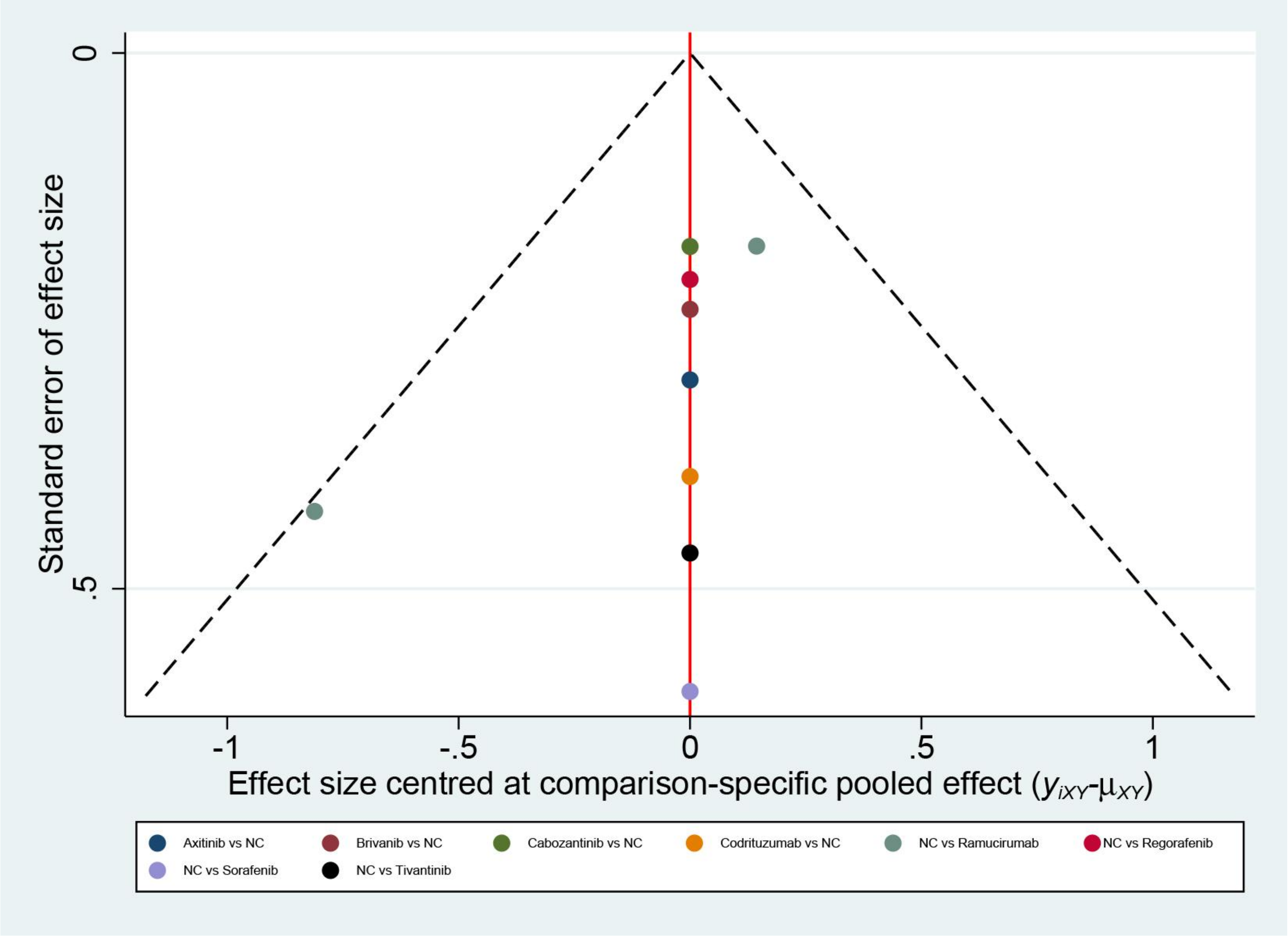

Figure S4. Publication bias testing by funnel plot regarding progression-free survival of patients without previous systematic therapy.

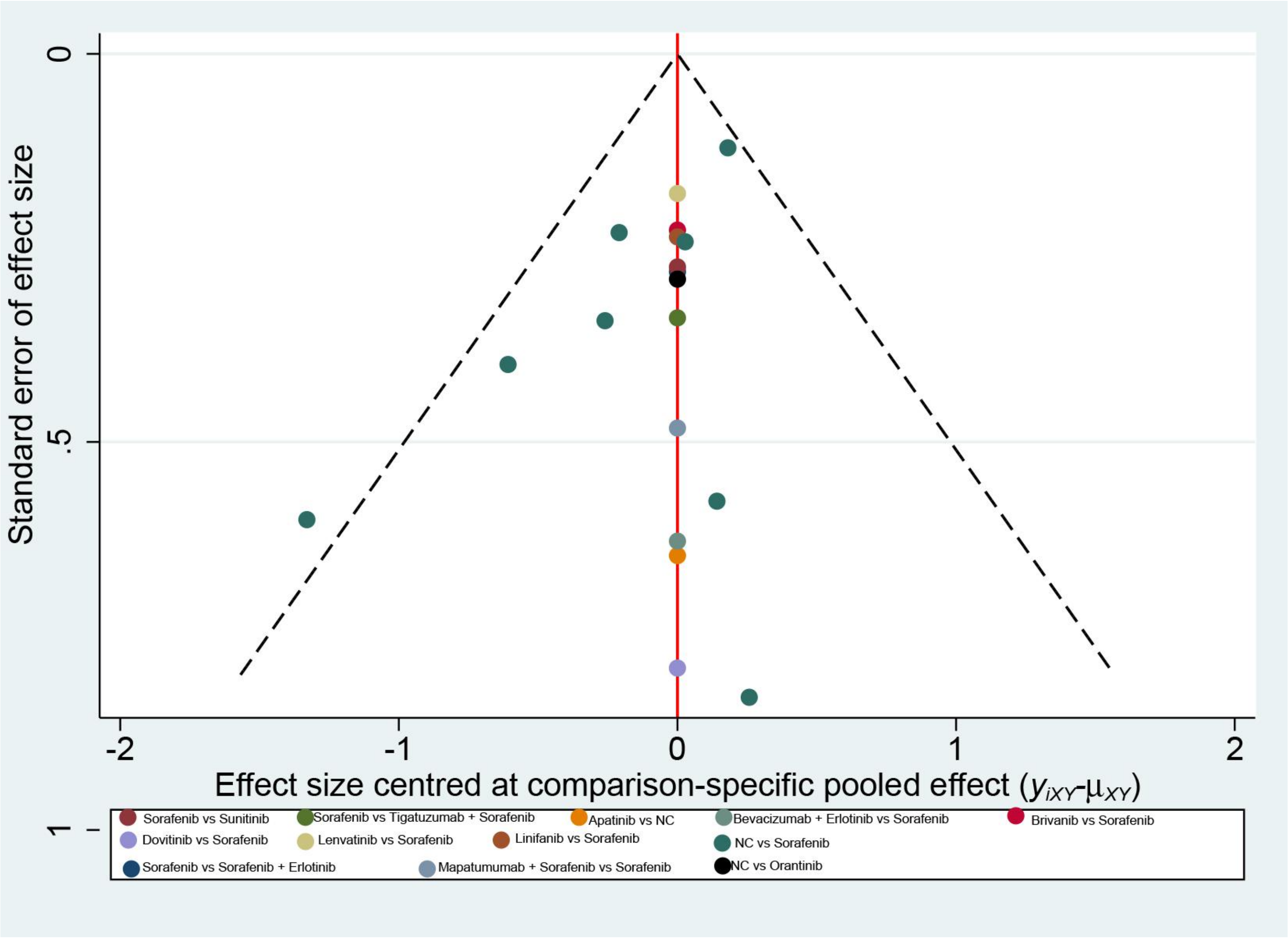

Figure S5. Publication bias testing by funnel plot regarding progression-free survival of patients with previous systematic therapy.

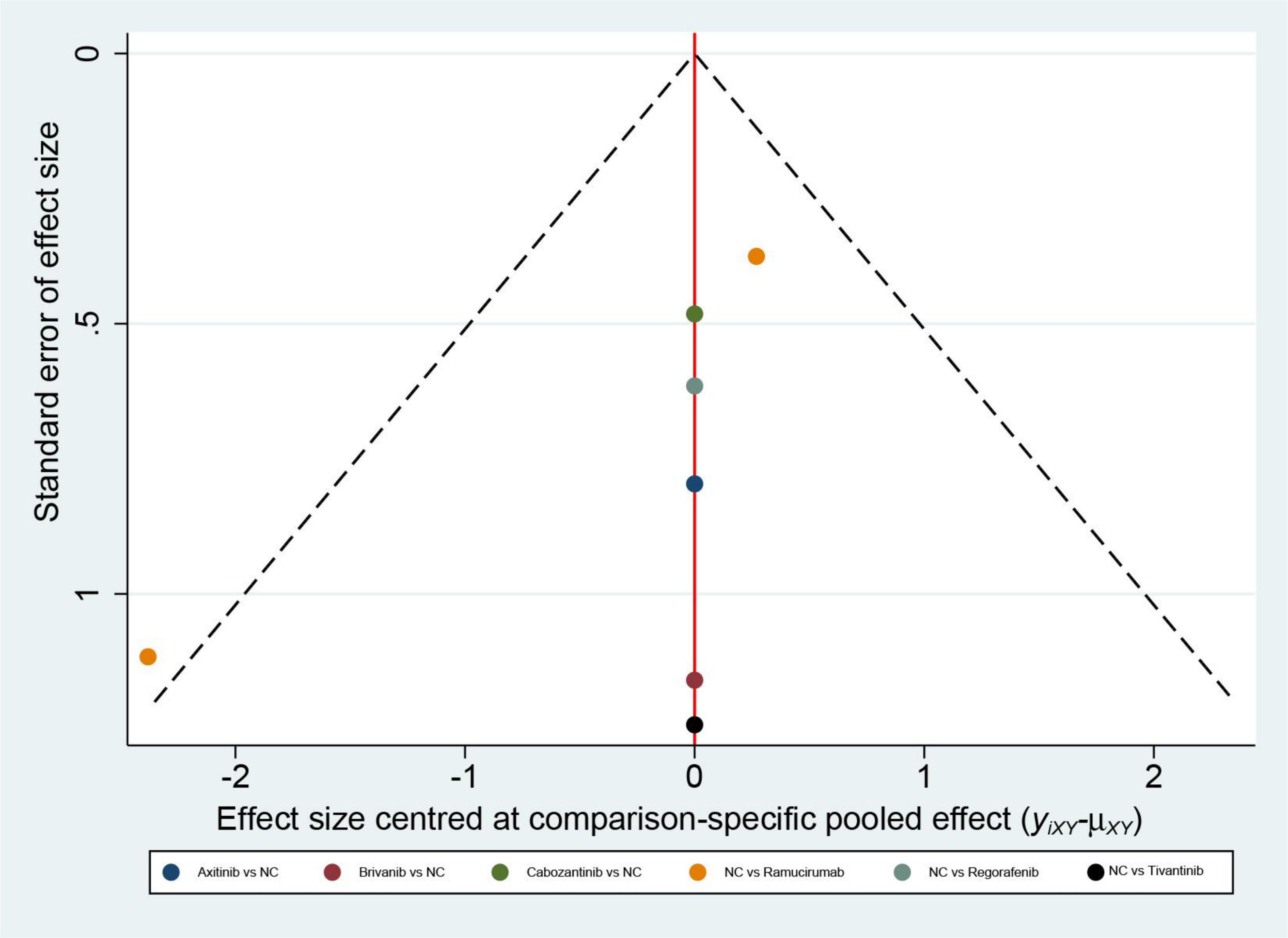

Figure S6. Publication bias testing by funnel plot regarding adverse event of patients without previous systematic therapy.

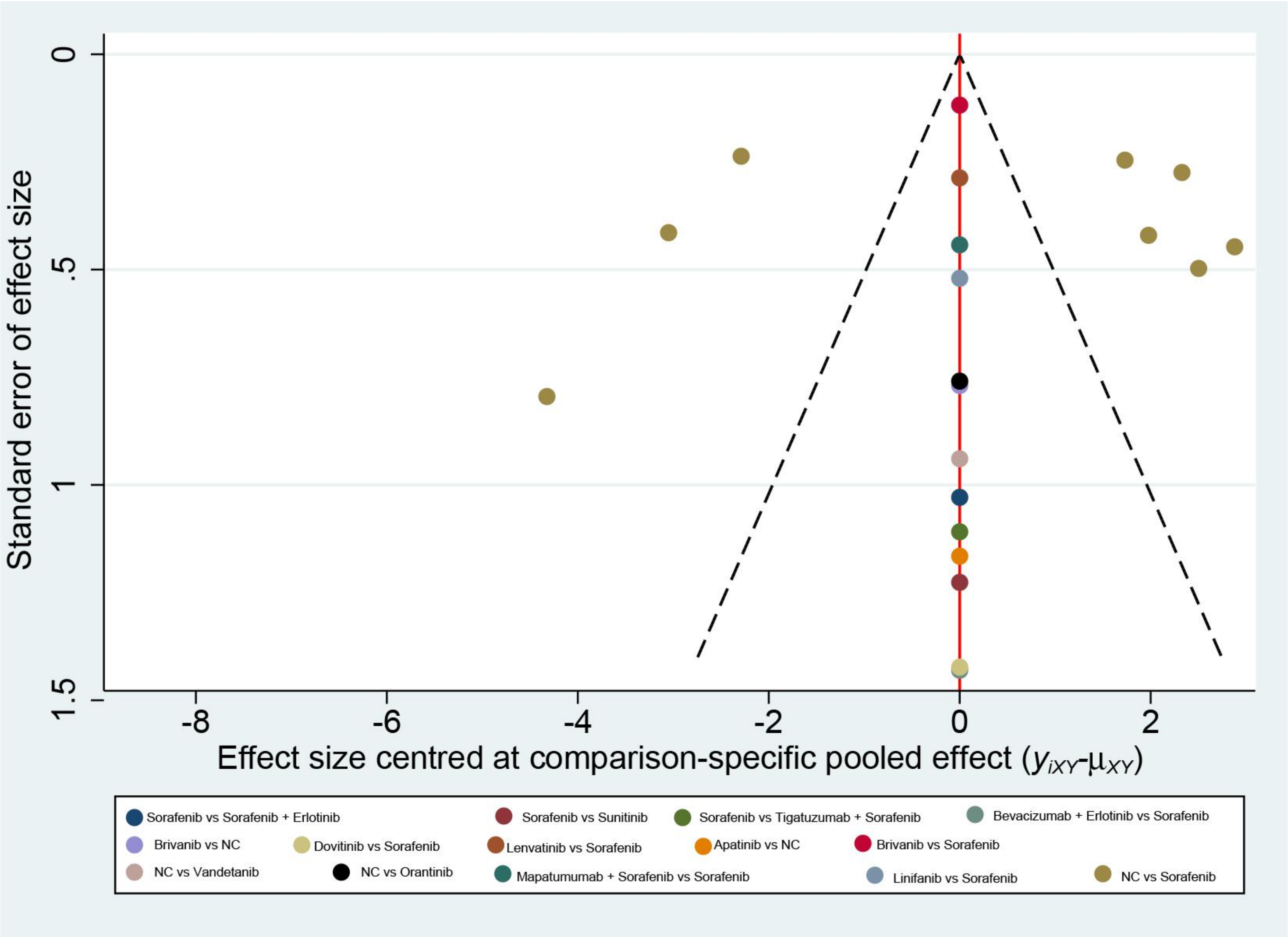

Figure S7. Publication bias testing by funnel plot regarding adverse event of patients with previous systematic therapy.

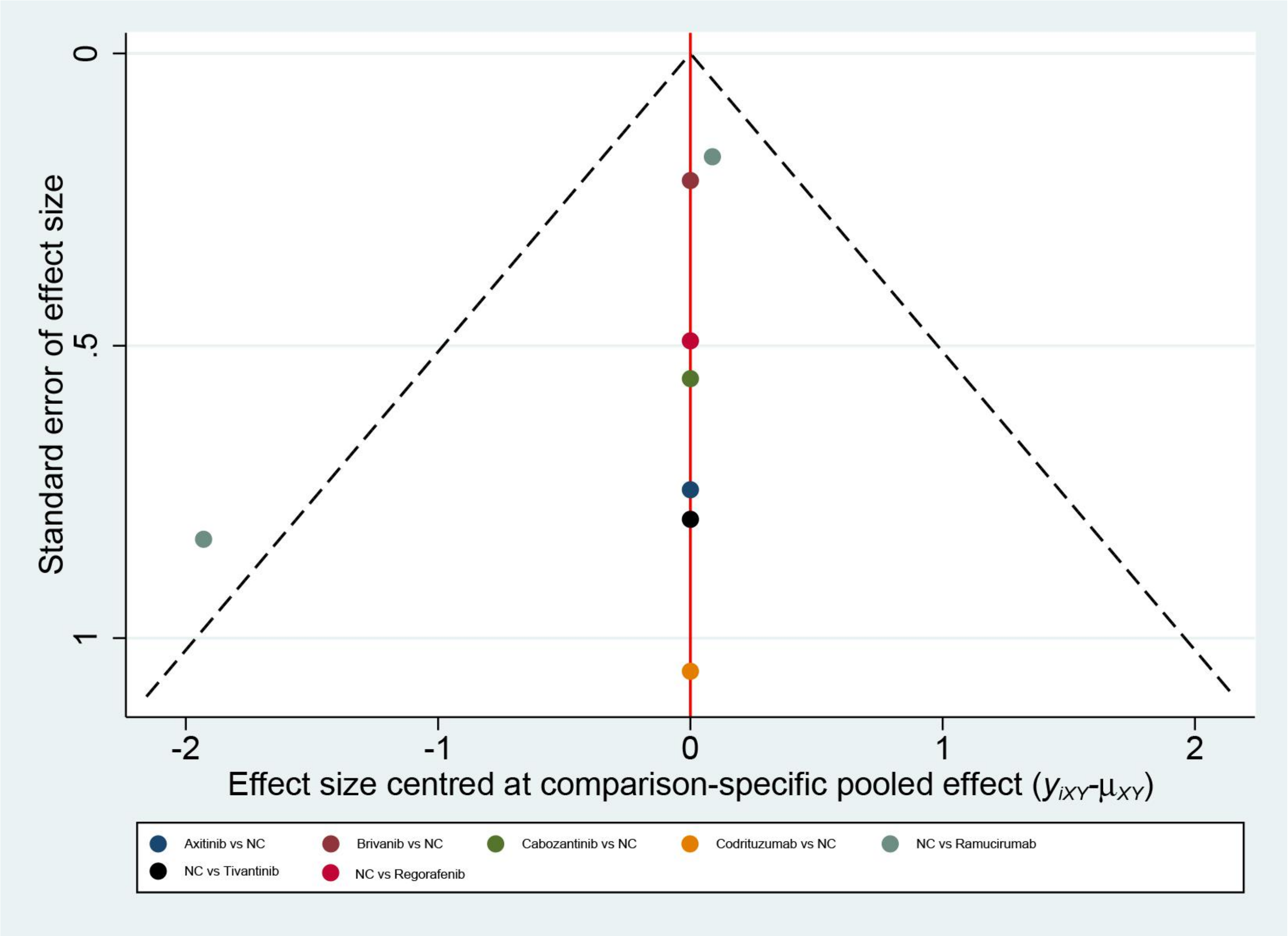

Supplement: Supplementary file 1 — Supplementary figures and tables. [file jcav10p4671s1.pdf]
